# Hippocampal surface analysis using discrete Ricci flow and Riemannian metrics for the diagnosis of Alzheimer’s disease

**DOI:** 10.21203/rs.3.rs-10382441/v1

**Published:** 2026-09-18

**Authors:** Fatemeh Ahmadi, Behroz Bidabad

**Affiliations:** 1 Department of Mathematics and Computer Science, Amirkabir University of Technology (Tehran Polytechnic), Tehran, Iran.

**Keywords:** Alzheimer’s Disease, Hippocampus, Discrete Ricci Flow, Covariance Matrix, Affine Invariant Riemannain Metric (AIRM), Machine Learning

## Abstract

Alzheimer’s disease (AD) is a neurodegenerative disorder that is characterized by structural changes of the hippocampus, thus hippocampal morphology becomes an important biomarker for early diagnosis. We propose a geometry-aware framework for AD classification based on discrete Ricci flow and Symmetric Positive Definite (SPD) manifold learning. We conformally parameterize the hippocampus surface meshes from the MRI data using Ricci flow and retrieve three geometric descriptors, Heat Kernel Signature, Area Distortion and Conformal Factor, during the Ricci energy optimization process. These features are represented as covariance matrices, resulting in SPD descriptors per subject. To preserve intrinsic manifold structure, classification is performed by KNN with multiple SPD-aware metrics, including the Affine-Invariant Riemannian Metric (AIRM), Wasserstein Metric, Log-Euclidean Metric and so on. Experimental results on ADNI dataset show that AIRM achieves the best performance with 96% accuracy, which outperforms other metrics evaluated. The results demonstrate that the combination of geometric analysis based on Ricci flow and manifold learning is effective for the diagnosis of Alzheimer’s disease.

## 1 Introduction

Alzheimer’s disease (AD) is the most prevalent neurodegenerative disease and a leading cause of dementia worldwide. It is characterized by progressive mental deterioration, memory loss and structural brain atrophy. Early diagnosis of AD is important so that appropriate intervention can be made to slow the progression of the disease. One of the affected brain regions is the hippocampus which is of particular importance because it is one of the first anatomical structures to show morphological degeneration during the course of the disease. Therefore, the shape analysis of hippocampus has been used increasingly as an important biomarker for AD diagnosis [1].

Magnetic Resonance Imaging (MRI) is a non-invasive technique for examining structural change in the brain and has been widely used in studies of AD. Most traditional MRI-based methods are mainly concerned with volume measurements of brain regions. However, volumetric analysis may not capture subtle geometric deformations which may contain discriminative pathological information. Thus, the surface-based analysis methods have been gaining more and more attention since they allow to study the local geometric and topological variations of brain structures [2].

Some recent studies have explored different geometric settings for the analysis of hippocampal shape. In [3], the authors introduced Teichmuller shape descriptors for AD diagnosis and demonstrated that conformal geometry yields robust shape representations. A hyperbolic Wasserstein distance was proposed in [4] to compare anatomical structures on non-Euclidean manifolds. Another study for structural brain analysis was based on Tutte’s embedding and harmonic mapping with KNN classification [5]. More recently, deep learning approaches such as surface-based transformer frameworks [6] have demonstrated strong performance of the brain surface analysis in AD classification. Though these methods can achieve high classification accuracy, they typically need large training datasets and might not be interpretable.

Riemannian geometry-based learning on Symmetric Positive Definite (SPD) matrices has become a useful alternative for learning through covariance representations. SPD matrices automatically capture second-order statistical relationships while keeping important structural details that help in distinguishing different cases [7].

A key part of classification on non-Euclidean spaces is selecting the right distance metric. When feature representations exist on Riemannian manifolds, like the space of Symmetric Positive Definite matrices, using regular Euclidean distance doesn’t properly show the real shape or structure of the data. Because of this, many different metrics that take into account the structure of data have been developed for use in classification tasks, especially when working with K-Nearest Neighbor (KNN) classifiers.

One of the most common ways to measure distances on the SPD manifold is the Affine-Invariant Riemannian Metric, which helps calculate distances that don’t change when the data undergoes certain transformations. This measure has been thoroughly researched for use with covariance-based methods and has shown good results in biomedical and computer vision areas, which we applied in our earlier work [8]. However, its computational cost can be high because it involves matrix logarithm and exponential operations.

To deal with computing challenges, the Log-Euclidean Metric (LEM) was created as a faster way to handle SPD matrices. It uses matrix logarithms to move these matrices into a simpler Euclidean space, making it easier to calculate distances while still keeping the important geometric traits. LEM is commonly used in classification systems that use covariance-based features because it offers a good mix of speed and accurate geometric representation.

Besides Riemannian metrics, distances based on optimal transport have become more popular. The Wasserstein metric offers a good method to compare how different probability distributions are and has been adapted for use with SPD matrices to analyze shapes and brain images [4]. Unlike classical metrics, Wasserstein distance takes into account both the position and the distribution of data, which makes it a good choice for comparing shapes in anatomy.

For comparison purposes, Euclidean distance in the regular matrix space is commonly used because it’s simple, but it doesn’t take into account the special structure of SPD matrices and might result in less accurate classification. Tangent space projection methods work by approximating the manifold in a small area around a specific point. They do this by transforming SPD matrices into a Euclidean space that touches the manifold at that point, which makes it possible to use regular machine learning techniques [9].

Recent research shows that certain metrics that take into account the structure of data, like the Affine-Invariant Riemannian Metric, Log-Euclidean Metric, and Wasserstein distance, work better than traditional methods based on Euclidean geometry in tasks related to biomedical imaging and shape analysis. This improvement has been seen in both traditional SPD-based learning methods and newer deep Riemannian neural networks, where keeping the natural geometry of the data’s underlying structure helps create better distinguishing features [10, 11].

In this paper, we introduce a framework that uses geometry-aware methods to classify Alzheimer’s disease. This approach focuses on analyzing the surface of the hippocampus, a key brain structure affected by the disease. We use two main techniques: discrete Ricci flow for shaping and simplifying the surface data, and SPD manifold learning to find patterns in the data. These methods help improve the accuracy of disease classification. The surfaces of the hippocampus, taken from MRI data using FreeSurfer, are repeatedly reshaped using Ricci flow to create conformal mappings that keep the original geometric features intact. During the optimization process, several geometric features like Heat Kernel Signature (HKS), Area Distortion, and Conformal Factor are collected to describe how the surface shape changes over time. These features are shown using covariance matrices, creating SPD descriptors for each step in the optimization process.

To keep the natural geometry of the covariance descriptors, classification is done using K-Nearest Neighbor (KNN) with several metrics that are aware of SPD structures, such as AIRM, Log-Euclidean, Wasserstein, Euclidean, and Tangent Space Representation. The experimental results on the ADNI dataset show that the proposed framework performs well and shows how effective Riemannian geometry is at capturing small changes in the hippocampus related to Alzheimer’s disease.

The main contributions of this work can be summarized as follows:

- A new method that uses discrete Ricci flow and covariance-based SPD representation to analyze the surface of the hippocampus.
- Extracting dynamic geometric features during Ricci flow optimization to track how the surface changes over time.
- A comparison of several manifold-aware metrics used for classification based on symmetric positive definite matrices.
- Show how Riemannian manifold learning works well for diagnosing Alzheimer’s disease.

The rest of this paper is arranged in the following way. Section 2 introduces the suggested framework, which covers the mathematical basis, the discrete Ricci flow method, the process of extracting features, the use of covariance to represent data, and the classification approach based on manifolds. Section 3 talks about the results from the experiments and how well the system performed. Section 4 discusses how well the suggested method works. Finally, Section 5 wraps up the paper and talks about where future research might go.

## 2 Proposed Framework

In this study, a framework was created that takes into account the shape of the brain’s hippocampus. It uses covariance representations and a method called Riemannian manifold learning to classify the surfaces of the hippocampus. The whole process has four main steps: brain surface reconstruction from 3D MRI data using FreeSurfer, creating a parameterization of the hippocampal surface using Ricci flow, extracting covariance features in Ricci energy optimization, and doing classification based on Riemannian metrics.

The method starts by reconstructing the brain surface and extracting hippocampus surface meshes from 3D MRI data using FreeSufer, then using discrete Ricci flow on the surfaces of hippocampal meshes to parameterize the surfaces. Ricci flow is a way to change the shape of a surface by slowly adjusting its measurements based on how curved it is. The shape of a surface changes over time when it is subjected to Ricci flow, which is a process that gradually makes the curvature of the surface more even. Depending on the shape (called genus) of the surface, the geometry slowly becomes like a standard shape: a round ball for genus 0, a flat donut shape for genus 1, and a curved surface that’s like a saddle for genus 2 or more.

**Figure 1:**
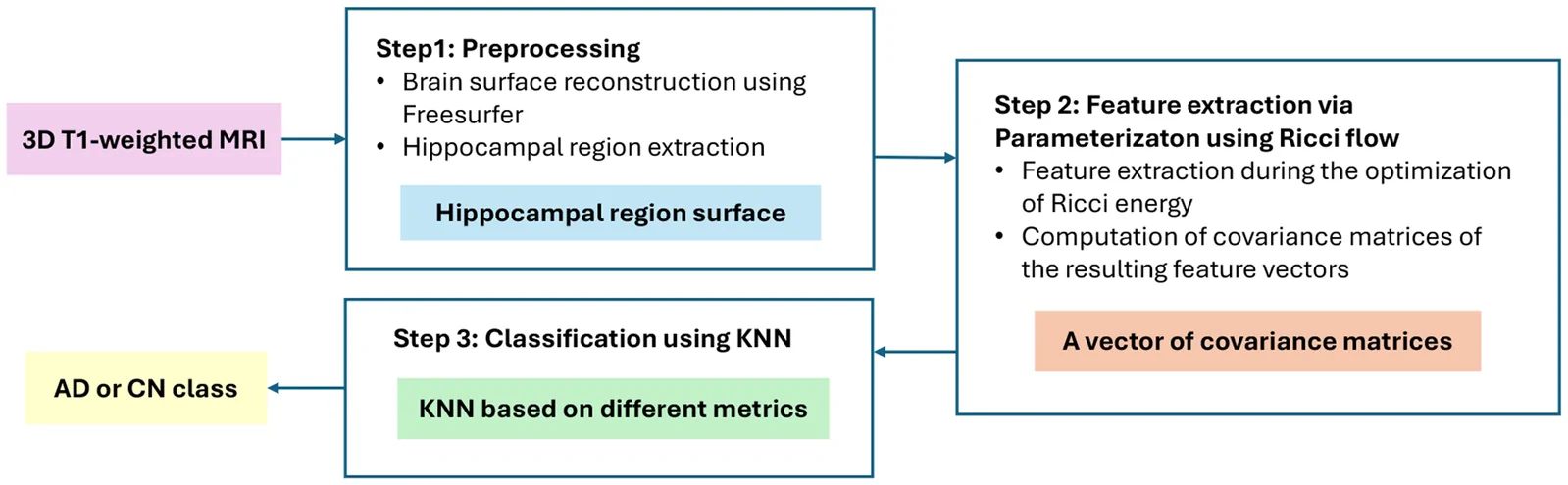
Block diagram of the proposed method in this paper.

During the Ricci flow process, the surface of the hippocampus is mapped in a way that keeps its natural shape and structure intact. At each step of the Ricci energy optimization process, the surface shape changes to move closer to a standard parameterization. In this study, certain optimization steps were used, chosen through trial and error, to show how the shape of the hippocampal surface changes gradually as the Ricci flow process runs. Figure 1 shows the complete block diagram of the method we suggest.

### 2.1 Discrete Ricci Flow and Surface Parameterization

Ricci flow is a way to study shapes in geometry, developed by Hamilton, which changes the shape of a space based on how curved it is. The main idea of Ricci flow is to gradually make the shape of a surface smoother by spreading out the curvature across the whole surface, just like how heat spreads out over time. Areas that have a lot of curve get smaller over time, and areas with less curve get bigger, which makes the overall curve more even across the whole shape.

Let *g_ij_* represent the Riemannian metric tensor and *K* stand for the Gaussian curvature. The smooth Ricci flow is defined as

(1) $$\frac{dg_{ij}(t)}{dt}=-2K(t)g_{ij}(t),$$

where *t* is the time parameter.

Ricci flow is often used for conformal surface parameterization because it keeps the original geometric features intact while minimizing changes to the shape’s measurement. In brain surface analysis, this feature allows for useful comparisons between different anatomical structures and helps in identifying unique geometric characteristics.

Because brain surfaces made from MRI data are shown as triangular meshes, the continuous Ricci flow method needs to be adapted for use with these discrete surfaces. Let *M* = (*V, E, F*) be a triangular mesh, where *V* , *E*, and *F* are the sets of vertices, edges, and triangular faces, respectively. The *discrete Gaussian curvature* at a vertex *v_i_* is the angle deficit:

(2) $$K\left(v_{i}\right)=\{\begin{array}{ll} \pi -\sum_{j}\theta_{j}, & \text{for boundary vertices,}\\ 2\pi -\sum_{j}\theta_{j}, & \text{for interior vertices,} \end{array}$$

where *θ_j_* are the angles incident to vertex *v_i_*.

In this paper, we develop our discrete Ricci flow framework based on three fundamental concepts, i.e., initial circle packing, Ricci energy optimization, and planar embedding [12].

#### 2.1.1 Initial Circle Packing

The first step of Ricci flow is to build an initial circle packing metric on the triangular mesh. Circle packing is a discrete model of conformal geometry in which a circle is associated to each vertex of the mesh. Different circle packing schemes can be defined based on the geometric relationship between neighbouring circles, such as Thurston, tangential and inversive distance circle packing [13]. We use inversive distance circle packing in this work because it is flexible enough to represent general surface geometries.

For each vertex *v_i_*, the *circle radius* $\gamma_{i}^{jk}$ is initialized using neighboring edge lengths:

(3) $$\gamma_{i}^{jk}:=\frac{l_{ij}+l_{ki}-l_{jk}}{2},$$

where *l_ij_* denotes the edge length between vertices *v_i_* and *v_j_*. The *inversive distance η_ij_* between neighboring circles is computed as

(4) $$\eta_{ij}:=\frac{l_{ij}^{2}-\gamma_{i}^{2}-\gamma_{j}^{2}}{2\gamma_{i}\gamma_{j}}.$$

This circle packing metric is used as the initial state for the optimization process via Ricci flow.

#### 2.1.2 Ricci Energy Optimization

Next, we optimize the conformal metric via the Ricci energy minimization after we construct the initial circle packing metric. The Ricci energy, measures how far the current curvature distribution is from the target curvature distribution.

We refer to *ui* = log *γ_i_* as the conformal factor for the vertex *v_i_*. *The Ricci energy* is given by

(5) $$E(u):=\int \underset{i}{\sum }\left({\bar{K}}_{i}-K_{i}\right)du_{i},$$

where $\bar{K}$ and *K* are respectively the target and current curvatures.

In order to optimize Ricci energy the edge weights are calculated using the power centers of adjacent triangles, a method for calculating the power center of a triangle is discussed in the our previous work [14]. Based on these edge weights the *Hessian matrix* is built:

(6) $$\frac{\partial^{2}E}{\partial u_{i}\partial u_{j}}=\{\begin{array}{ll} \sum_{k}w_{ik}, & i=j,\\ -w_{ij}, & e_{ij}\in E,\\ 0, & \text{otherwise.} \end{array}$$

The conformal factors are updated iteratively with the Newton’s method:

(7) $$\delta u=H^{-1}(\bar{K}-K),$$

where *H* is the Hessian matrix. Optimization proceeds until the curvature error reduces below a defined tolerance. During optimization, the mesh metric is transformed toward the target curvature.

#### 2.1.3 Planar Embedding

After optimizing the Ricci energy, the resulting metric is placed into the Euclidean plane to create a two-dimensional conformal parameterization of the surface. The embedding process tries to keep the original shapes and distances between nearby points, while making the surface flat. Initially, a triangular shape is chosen and embedded isometrically in the plane:

(8) $$\phi \left(v_{0}\right)=0,\;\phi \left(v_{1}\right)=l_{01},\;\phi \left(v_{2}\right)=l_{20}e^{i\theta_{0}^{12}},$$

where *ϕ* denotes the embedding function. The intersection points of circles centered at previously embedded vertices are used to recursively embed neighboring faces. This process goes on until every face of the mesh lies flat in the same plane.

### 2.2 Feature Extraction During Ricci Energy Optimization

To understand how the shape of the hippocampal surface changes during the Ricci flow process, three different geometric features were collected at certain points during the optimization steps:

- Heat Kernel Feature:
  The heat kernel shows how heat spreads over a surface and helps understand the shape’s natural geometry and structure. It is commonly used to study and describe the surface’s features. Heat Kernel Signature (HKS) comes from the Laplace–Beltrami operator, which means it shows the true shape of a surface and doesn’t change if the surface is stretched or bent without stretching. In this study, HKS is used to analyze the shape features of the hippocampal surface meshes.
  *Heat diffusion* on a Riemannian manifold *M* is governed by the heat equation:
(9) $$\Delta_{g}u(x,t)=-\frac{\partial u(x,t)}{\partial t},$$
  where Δ_*g*_ denotes the Laplace–Beltrami operator and *u*(*x, t*) is the heat distribution at time *t*.
  The Laplace-Beltrami operator is used to construct separate triangular meshes, which are constructed using weights based on the cotangent of their edges. This results in *L* being a discrete Laplatic matrix that can be divided into eigenvalues and corresponding vertices.
(10) $$L=\Phi \Lambda \Phi^{T},$$
  where Λ and Φ denote the eigenvalues and eigenvectors of *L*, respectively. The heat kernel is then computed as
(11) $$HK(t)=\Phi e^{-\Lambda t}\Phi^{T}.$$
  The Heat Kernel Signature is defined using the diagonal elements of the heat kernel:
(12) $$HKS(x,t)=HK_{t}(x,x).$$
  Local surface geometry is represented in multi-scale, compact representation using HKS. Smaller values of *t* represent only local geometric details, while larger values represent more global shape information.
- Area Distortion:
  Area distortion measures how much a surface stretches or shrinks in specific areas during the conformal parameterization process. It gives details about how the shape is changing geometrically, as shown in the equation (13).
(13) $$AD(v)=\underset{t\in B}{\sum }\mathrm{area}(t)-\underset{t\in B}{\sum }\hat{\mathrm{area}}(t),$$
  where *area*(*t*) represents the area of triangle *t* on the initial mesh, $\hat{\mathrm{area}}$ denotes the area of triangle *t* on the mesh in the current stage, and *B* refers to the one-ring neighborhood of vertex *v*.
- Conformal Factor:
  The conformal factor shows how the shape of the hippocampal surface changes in small areas during Ricci flow, and it shows how the curvature affects the geometry of that surface. In the Ricci flow process, the conformal factor at the vertex *v_i_* is defined as *u_i_* = log(*γ_i_*), where *γ_i_* represents the radius of the circle at that vertex. By definition, *u_i_* shows the natural features of a surface and does not change when the surface is bent without stretching.
  These three features together show both the intrinsic shape changes and the extrinsic shape changes of the surface as it evolves during the Ricci flow optimization.

### 2.3 Covariance Matrix Representation

For each step of optimization, the statistical connections between the extracted geometric features were represented using covariance matrices. Let the feature vector that is extracted at the optimization step t for subject s be defined as:

(14) $$f_{s,t}={\left[f_{1},f_{2},\ldots ,f_{d}\right]}^{⊤},$$

where *d* denotes the number of geometric features. The corresponding covariance matrix is then computed as:

(15) $$C_{s,t}\in R^{d\times d},$$

which is a symmetric positive definite (SPD) matrix capturing the statistical dependencies among the features.

Each subject is represented by a set of *T* SPD covariance matrices when *T* optimization steps are analyzed for each subject:

(16) $$\left\{C_{s,1},C_{s,2},\ldots ,C_{s,T}\right\}.$$

For the whole set of data including *N* individuals, the final form can be represented as a fourth-order tensor:

(17) $$X\in R^{N\times T\times d\times d},$$

where *N* represents the number of subjects, *T* refers to the number of optimization steps, and *d* is the count of extracted geometric features, each *d* × *d* matrix illustrates the covariance structure of the features at a specific optimization step.

### 2.4 SPD Manifold-Based Classification

Covariance matrices are symmetric positive definite matrices, which means they inherently exist on a Riemannian manifold rather than a Euclidean vector space. Therefore, the standard Euclidean distance method is not appropriate for precisely evaluating how similar different samples are.

To address this issue, K-Nearest Neighbor (KNN) classification was carried out using a variety of manifold-aware distance metrics defined on the SPD manifold, including:

- Euclidean Metric,
- Affine-Invariant Riemannian Metric (AIRM),
- Log-Euclidean Metric,
- Wasserstein Metric,
- Tangent Space Representation.

#### 2.4.1 Distance Metrics on the SPD Manifold

SPD matrices inherently exist on a Riemannian manifold instead of a Euclidean vector space. Therefore, traditional Euclidean distances might not adequately represent the true geometric structure of covariance matrices. To resolve this issue, multiple Riemannian and manifold-sensitive metrics were used for K-Nearest Neighbor (KNN) classification.

- Euclidean Metric
  The Euclidean metric considers symmetric positive definite matrices as regular matrices in Euclidean space and calculates the Frobenius norm between them:
(18) $$d_{E}(A,B)=|A-B{|}_{F}.$$
  Although it is computationally efficient, the Euclidean metric does not take into account the curved geometry of the SPD manifold. As a result, this approach could result in lower accuracy in classification when compared to using Riemannian metrics.
- Affine-Invariant Riemannian Metric (AIRM)
  The Affine-Invariant Riemannian Metric (AIRM) is among the most commonly used distance measures on the SPD manifold. It calculates the geodesic distance between two symmetric positive definite matrices while maintaining affine invariance. Given two symmetric positive definite matrices (A) and (B), the AIRM distance is defined as
(19) $$d_{AIRM}(A,B)={\left|log(A^{-1/2}BA^{-1/2})\right|}_{F},$$
  where (log(⋅)) is applied to a matrix and $\left(|\cdot {|}_{F}\right)$ or Frobenius norm is used to measure the magnitude of a matrix. AIRM acknowledges the inherent geometric structure of manifolds and offers strong ability to distinguish between different covariance-based representations. However, the process is computationally expensive because it involves repeatedly performing matrix logarithm and matrix square-root calculations.
- Log-Euclidean Metric
  The Log-Euclidean metric maps SPD matrices to the tangent Euclidean space by taking the matrix logarithm, which simplifies computations on the SPD manifold. The distance between two SPD matrices is given by
(20) $$d_{LE}(A,B)=|log(A)-log(B){|}_{F}.$$
  This approach preserves important geometrical features and drastically reduces the computational burden in comparison to AIRM. The calculations are done in the log domain so the Log-Euclidean framework gives a practical and effective way to approximate the geometry of the manifold.
- Wasserstein Metric
  The Wasserstein metric, most often called the Earth Mover’s Distance, originates from optimal transport theory, and has received increasing attention in the SPD matrix analysis field. That is mathematical way to measure how different two sets of data are, based on the “work” it would take to change one set into the other. This metric measures transport cost between covariance distributions and is often very numerically stable. The Wasserstein metric can provide smoother geometric behavior than AIRM, while retaining meaningful manifold-aware distances.
  For two SPD matrices (A) and (B), the Wasserstein distance is defined as
(21) $$d_{W}(A,B)=\sqrt{tr\left(A+B-2{\left(A^{1/2}BA^{1/2}\right)}^{1/2}\right)},$$
  where the notation tr denotes the trace of the matrix.
- Tangent Space Representation
  To reduce the computational complexity, the SPD matrices can be projected into the tangent space of the manifold. This is achieved by the matrix logarithm operation:
(22) T=log(A).
  The upper triangular part of the tangent matrices is vectorized after projection and considered as Euclidean feature vectors. Then, standard machine learning classifiers, such as KNN, can be directly applied in Euclidean space.
  The tangent space representation offers significant computational speedup while preserving much of the discriminative manifold structure. Therefore, it is widely used in SPD-based machine learning and neuroimaging applications.

Distances over T steps of Ricci flow optimization were calculated for each pair of subjects by averaging (mean, max, min, or median) distances between the corresponding covariance matrices. These geometry-aware similarity measures were then used in KNN classification of Alzheimer’s disease and healthy control subjects.

**Figure 2:**
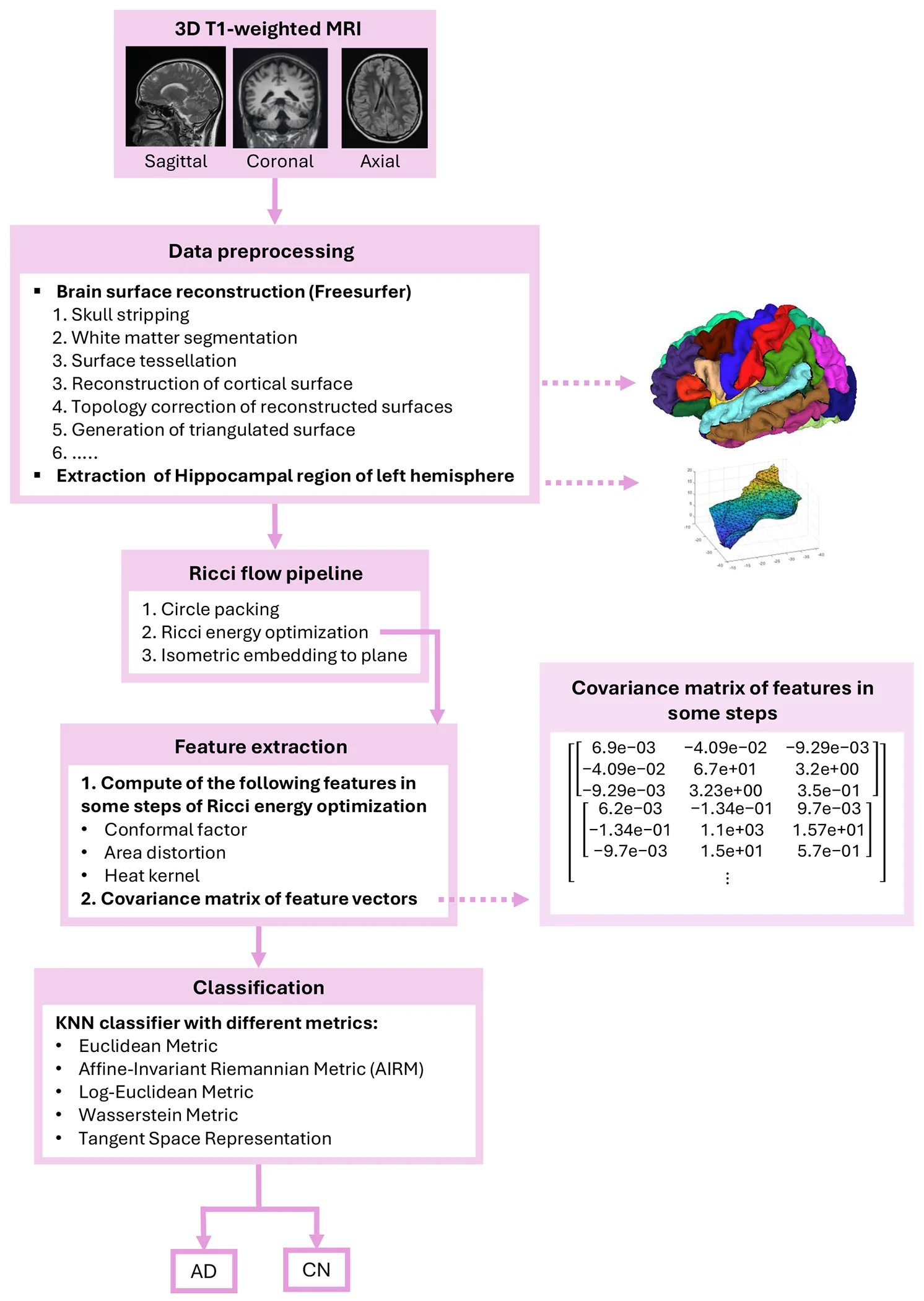
Graphical diagram of the proposed method in this paper.

Therefore, the proposed framework integrates geometric brain surface analysis, covariance representation learning and Riemannian manifold classification to detect subtle structural changes in hippocampal morphology associated with neurodegenerative changes. A block representation of the proposed technique is shown in Figure 2. The figure shows different components and their interaction. Algorithm 1 presents the algorithm that defines this process and provides a step-by-step framework for effective implementation of the technique. The block diagram along with the algorithm are designed to clarify the methodology and to make the approach presented in this work easy to understand.

**Algorithm 1:** Alzheimer’s disease diagnosis using Ricci flow and manifold-based SPD classification

**Input:** 3D T1 Weighted MRI data.
**Output:** Predicted class label (AD or CN).
1. Apply the FreeSurfer automated processing pipeline to reconstruct the cortical surface triangulation mesh from MRI data.
2. Extract the hippocampal region of the left hemisphere.
3. Normalize the hippocampal meshes by equalizing the number of vertices using down-sampling (or up-sampling).
4. Convert the hippocampal meshes into Delaunay triangulation meshes.
5. Detect the boundary vertices of each hippocampal mesh.
6. Compute the inversive distance circle packing metric for each mesh according to Section 2.1.1.
7. Optimize the Ricci energy using Newton’s method to obtain conformal factors according to Section 2.1.2.
8. Perform planar embedding of the optimized meshes according to Section 2.1.3.
9. For each optimization step *t* = 1, … , *T*:
(a) Compute the Heat Kernel Signature (HKS) on the hippocampal surface.
(b) Compute the Area Distortion feature.
(c) Compute the Conformal Factor feature.
(d) Construct the feature vector $f_{s,t}={\left[f_{1},f_{2},\ldots ,f_{d}\right]}^{⊤}$.
(e) Compute the covariance matrix **C**_*s,t*_ of the extracted features.
10. Represent each subject as a sequence of SPD covariance matrices: {**C**_*s*,1_, **C**_*s*,2_, … , **C**_*s,T*_}
11. Compute pairwise distances between subjects using SPD manifold metrics: AIRM, Log-Euclidean, Wasserstein, Euclidean, and Tangent Space Representation.
12. Perform KNN classification based on the computed manifold distances.
13. Assign the final class label (AD or CN) to the input MRI.

## 3 Methods

We showed the efficiency and effectiveness of our method by analyzing human brain cortices of Alzheimer’s disease (AD) and cognitively normal (CN) people. The computational tasks were performed in MATLAB and Python on a Windows 11 laptop with a 2.10 GHz 13th Gen Intel(R) Core(TM) i5-13420H and 16 GB RAM.

### 3.1 Dataset

This study was conducted using the data from the Alzheimer’s Disease Neuroimaging Initiative (ADNI) database [15] (https://adni.loni.usc.edu). The ADNI protocols were approved by all the Institutional Review Boards of the participating institutions. Only data from volunteers who had provided written informed consent were used to complete these analyses. All methods in this study were carried out in accordance with the relevant guidelines and regulations. De-identified data are available to qualified researchers who register and are approved for a data access application from ADNI.

The dataset collected from ADNI-1 and ADNI-2 consisting of 200 subjects including cognitively normal (CN) individuals and Alzheimer’s disease (AD) patients. The ADNI dataset contains longitudinal MRI scans acquired at multiple time points for many of the participants. For this study, only the baseline (first available) MRI scan of each subject was selected to ensure that each subject is represented by a single MRI volume. Table 1 describes the demographic characteristics of the participants in the study, including the number of subjects, sex distribution, and age statistics, which provide an overview of the study population.

### 3.2 Data preprocessing

**Figure 3:**
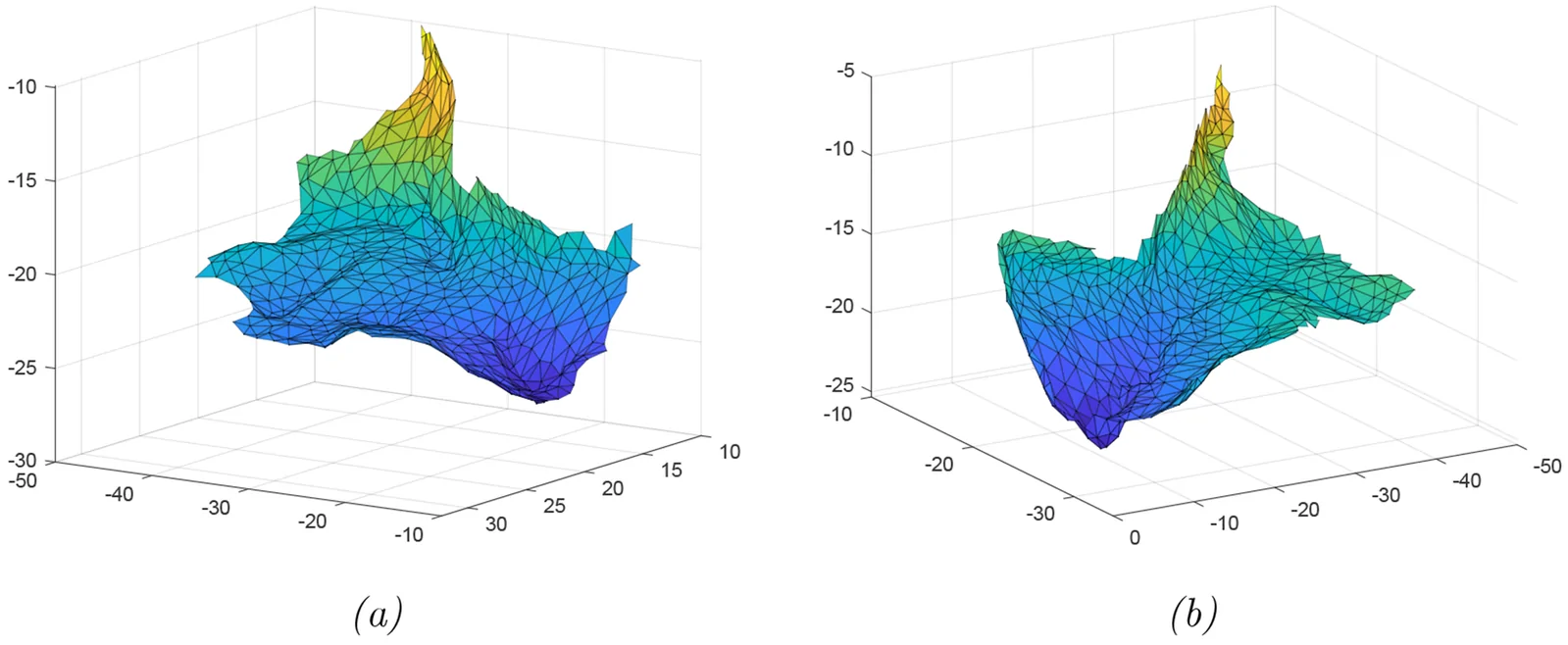
The hippocampal regions in the left cerebral hemispheres of two subjects, corresponding to (a) CN and (b) AD.

We applied the FreeSurfer automated processing pipeline as described in [16] for automatic skull stripping, tissue classification, surface extraction and parcellation of cortical and subcortical regions. The pipeline computes geometric features such as curvature, curving and local folding for each parcellation and offers data on surface and volume for about 34 different cortical structures. The hippocampal region on the surface of the left hemisphere was first obtained by parcellation. Figure 3 shows the hippocampal regions of left hemisphere of an AD subject and a CN subject.

### 3.3 Experimental

**Figure 4:**
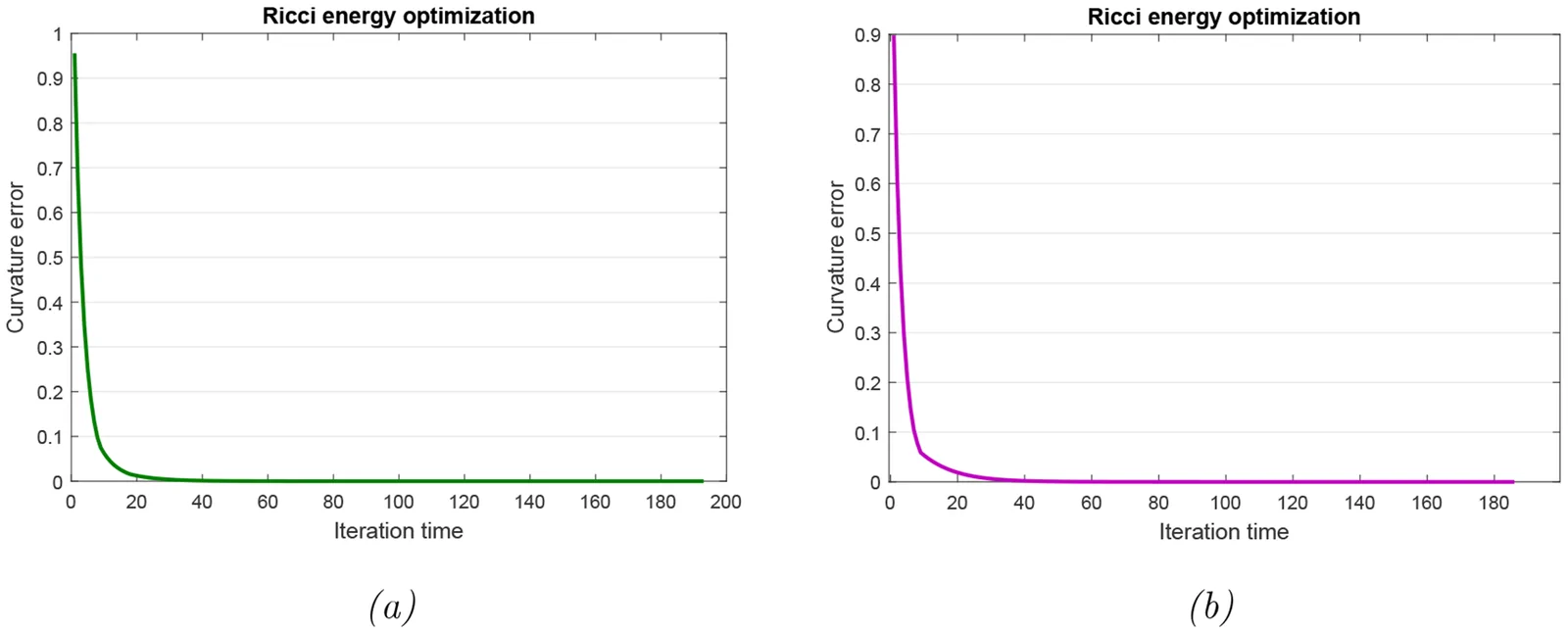
Optimization of Ricci energy in hippocampal regions of CN (a) and AD (b) subjects.

**Figure 5:**
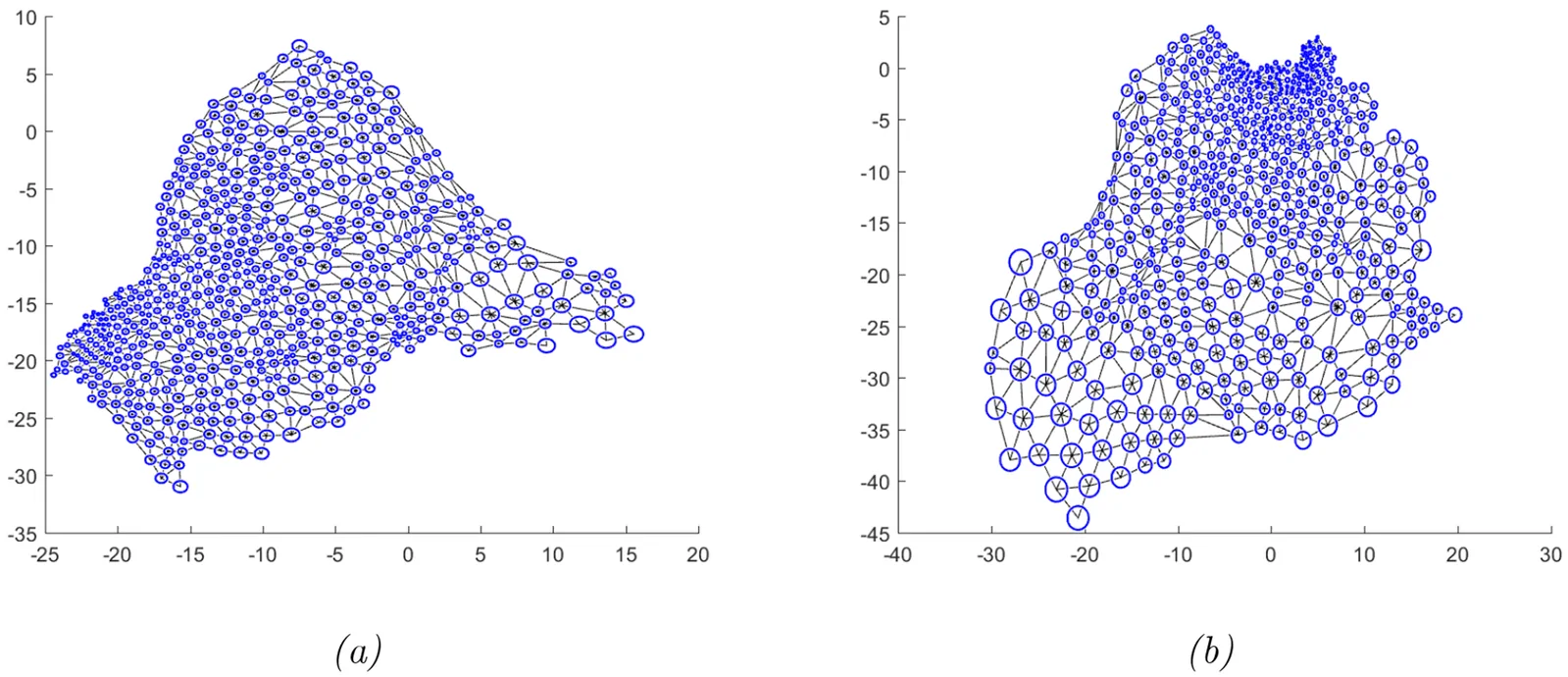
(a) and (b) show Planar embedding of hippocampal regions of CN and AD subjects, respectively, corresponds to inversive distance circle packing in the normalization space.

After extracting Hippocampal surface from brain cortex, each region was subjected to the Ricci flow method. Figures 4 and 5 show the Ricci energy optimization for each region and its planar embeddings, respectively.

**Figure 6:**
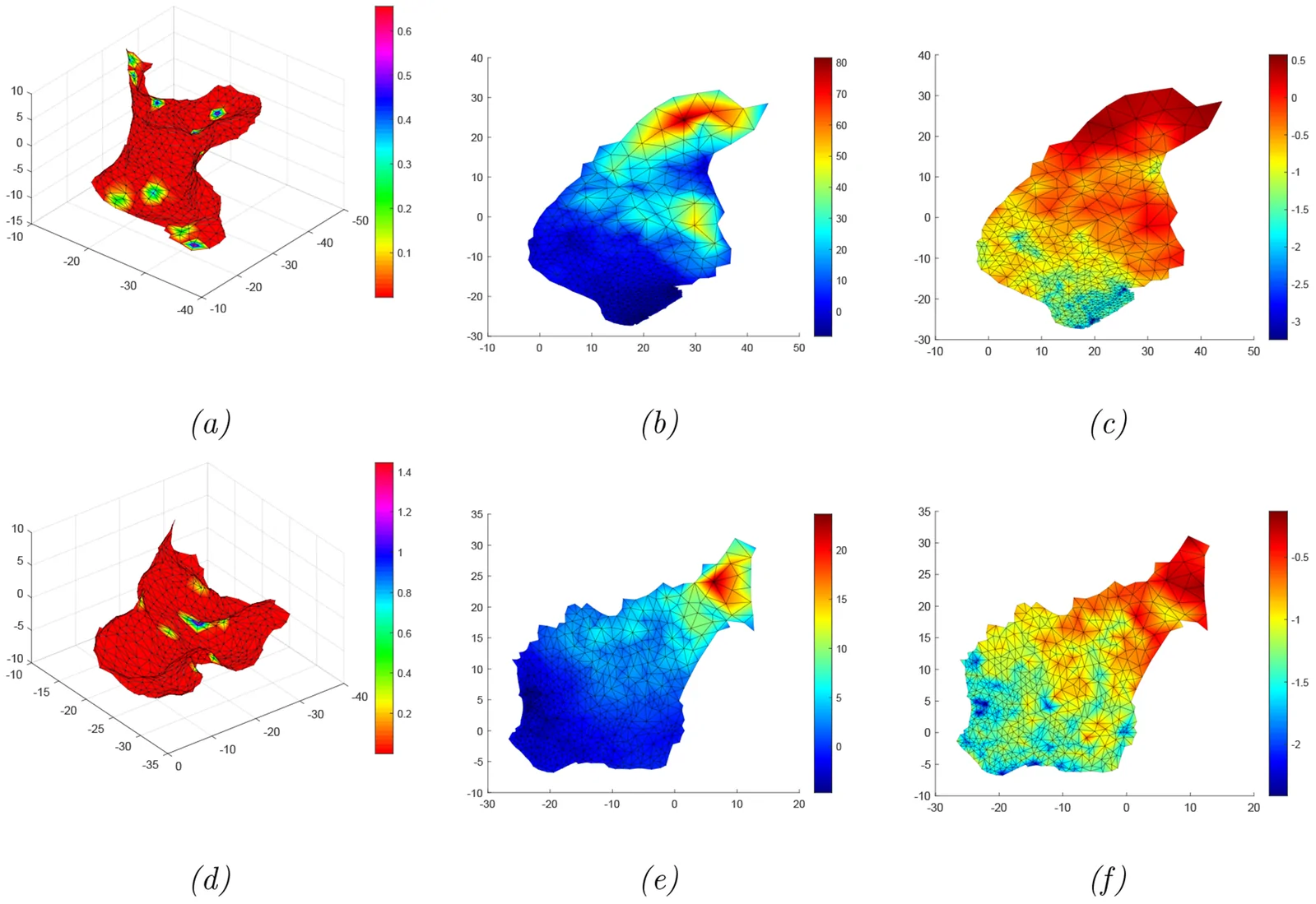
Distribution of features heat kernel, area distortion and conformal factor on hippocampus region of left hemisphere of an AD (a,b,c) subject and a CN (d, e, f) subject.

Then we computed the signatures of area distortion, heat kernel and conformal factor at T steps of Ricci energy optimization. Figure 6 displays the distribution of the features of area distortion and conformal factor on the triangulation mesh of the left hippocampal regions in the canonical domain space as well as the distribution of the feature heat kernel in a step of the Ricci energy optimization of the triangulation mesh of the left hippocampal regions of an AD subject and a CN subject. Obviously, each of these characteristics has a unique distribution in the hippocampal region.

For each optimization step the statistical relationships between the extracted geometrical features were modeled using covariance matrices. More specifically, we computed the covariance matrix from the three-dimensional feature vector consisting of: [Heat Kernel, Area Distortion, Conformal Factor]

Thus, each optimization step resulted in a symmetric positive definite (SPD) (3 × 3) covariance matrix. We considered 30 iterations of the Ricci flow for each subject (by trial and error). Thus, each subject was represented by a sequence of 30 SPD covariance matrices.

For all 200 subjects, the resulting representation can be expressed as a fourth-order tensor of size 200 × 30 × 3 × 3, where 200 is the number of subjects, 30 is the number of selected Ricci energy optimization steps, and each (3 × 3) matrix accounts for the covariance structure of the extracted geometric features.

To evaluate the performance of the proposed SPD-based framework, classification experiments were carried out first with the KNN classifier using different distance metrics, which include the Affine-Invariant Riemannian Metric (AIRM), Log-Euclidean Metric, Wasserstein Metric, Euclidean Metric and Tangent Space Representation. These metrics were chosen to investigate the effect of the different geometric interpretations of the SPD manifold on the classification performance.

Furthermore, these metrics were used to embed the SPD data into appropriate feature spaces, and the resulting representations were classified with Support Vector Machine (SVM), Logistic Regression (LR) and Extreme Gradient Boosting (XGBoost). This additional assessment provides an in-depth comparison of the proposed framework against both distance-based and conventional machine learning classifiers.

We evaluated the proposed framework on a dataset collected from ADNI (Table 1) consisting of 200 subjects including cognitively normal (CN) individuals and Alzheimer’s disease (AD) patients. Experiments were repeated 10 times with a random train-test split of 80/20. In each iteration 80% of the data was used for training and 20% for testing. The results in the following tables and figures are the mean±standard deviation of the performance indicators on the test sets over the 10 runs.

**Figure 7:**
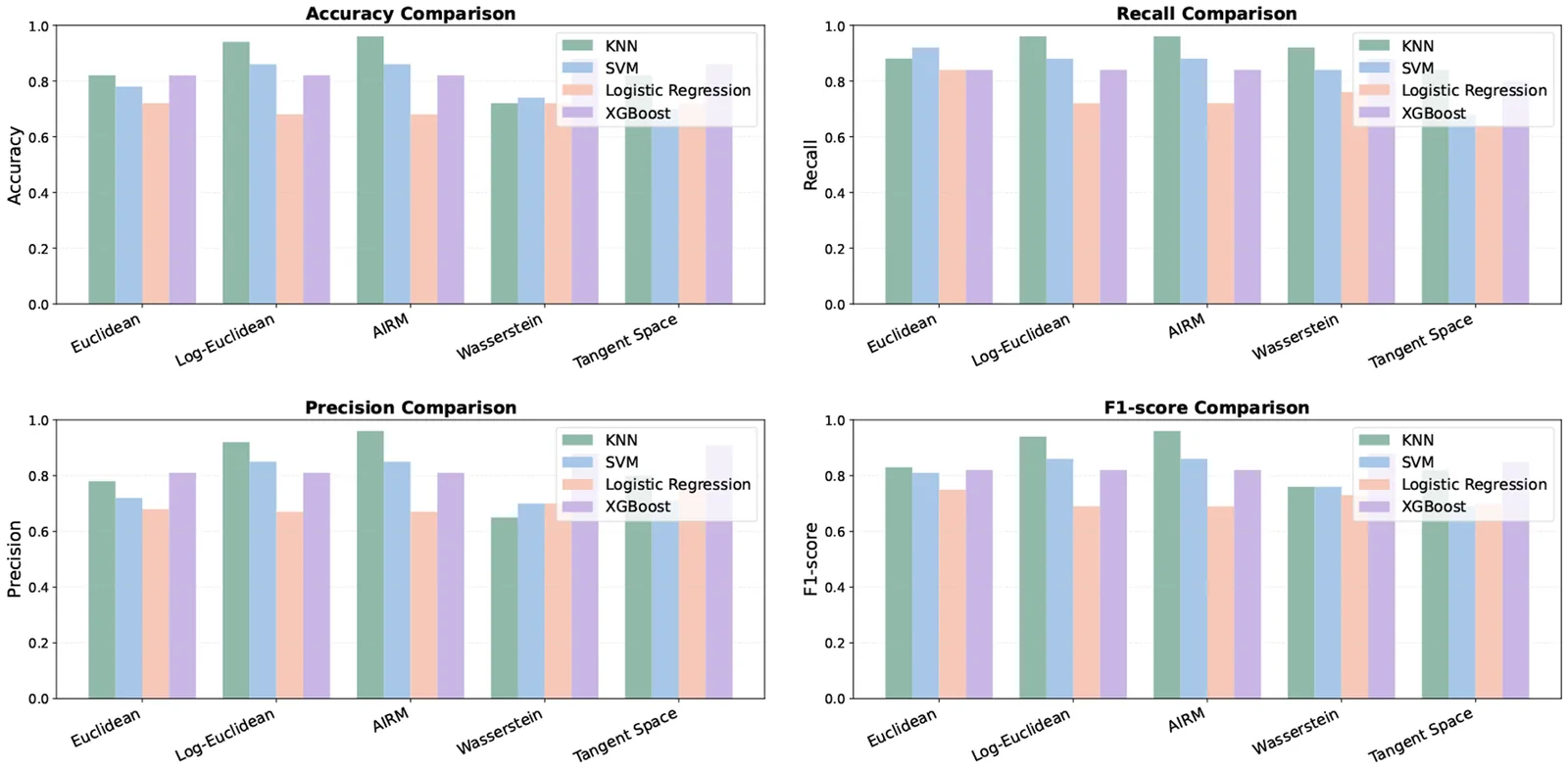
Classification performance of Euclidean and Riemannian SPD matrix representations using KNN, SVM, Logistic Regression, and XGBoost classifiers. Each subplot presents one evaluation metric (Accuracy, Precision, Recall, or F1-score), enabling a comprehensive comparison of the effectiveness of different manifold representations.

We present the quantitative results for all the metrics evaluated in Table 2 and Figure 7. Our observations show that manifold-aware metrics consistently outperform the Euclidean baseline, emphasizing the significance of preserving the intrinsic Riemannian structure of SPD matrices. The proposed method obtained the best performance under KNN classifier based on AIRM Metric with 96% accuracy among all the evaluated approaches. These measures are derived from the confusion matrix.

Confusion matrix is a common tool for evaluating the performance of the classification. It is a square matrix that compares the predicted class labels with the actual class labels (ground truth). Each row is the true label or actual class. Each column is the predicted label (class). Measures of recall, precision, accuracy, and *F*_1_ Score in equations (23) through (26) offer insight into a model’s performance on individual classes.

- **Accuracy:** The ratio of accurately predicted occurrences to all instances in the dataset is known as accuracy. It shows the overall accuracy rate of the classifier.
(23) $$\mathrm{Accuracy}=\frac{TP+TN}{TP+TN+FP+FN}.$$
- **Recall or True Positive Rate:** The percentage of real positives that are accurately detected is known as recall. It displays the model’s ability to recognize positive cases. Fewer false negatives are indicated by a strong recall.
(24) $$\mathrm{Sensitivity}=\frac{TP}{TP+FN}.$$
- **Precision (Positive Predictive Value):** Among all anticipated positives, precision measures the proportion of relevant or real positives among the chosen items. High precision emphasizes lowering false positives in classifications, meaning that more positively labeled images are in fact positive.
(25) $$\mathrm{Precision}=\frac{TP}{TP+FP}.$$
- *F*_1_ **Score:** In situations when there is an unequal distribution of classes, the *F*_1_ Score, which is a harmonic mean between precision and recall, provides a balance between the two. The *F*_1_ Score is especially helpful in determining the ideal ratio between recall and precision. Because it accounts for both false positives and false negatives, it is a better metric than accuracy when dealing with imbalanced datasets.
(26) $$F_{1}\mathrm{Score}=\frac{2*TP}{2*TP+FP+FN}.$$

As shown in Figure 7, the highest classification consistency was achieved by KNN based on AIRM Metric. This implies that AIRM can capture the intrinsic manifold geometry of the SPD matrices well and produce the most discriminative representation among the methods tested. Also, the results of KNN with Log-Euclidean Metric have shown good performance after AIRM. This means the logarithmic mapping preserves most of the geometric structure but some of the discriminative information may be lost.

Results on Wasserstein Metric, Euclidean Metric, and Tangent Space Representation were competitive for KNN and other classifiers. The XGBoost classifier with Wasserstein embedding performed best in this comparison.

The Tangent Space Representation performed in the middle. Mapping SPD matrices to a tangent space allows for easier computations but led to lower classification accuracy than direct manifold-based metrics like AIRM and LEM. The best performance in this comparison was achieved by XGBoost classifier with the Tangent Space Representation of features.

Finally, among all the metrics evaluated, the Euclidean Metric, was the least performing metric. This demonstrates the limitation of neglecting the non-Euclidean geometry of SPD matrices in measuring similarity.

The classification results verify the superiority of Riemannian-aware metrics, especially AIRM, in terms of the classification accuracy by preserving the intrinsic geometric relations in the SPD manifold.

Such results indicate that the use of Riemannian geometry in the classification framework provides a more discriminative representation of the covariance descriptors and thus increases the robustness and the classification accuracy when compared to the conventional Euclidean-based analysis.

#### t-Distributed Stochastic Neighbor Embedding (t-SNE):

t-Distributed Stochastic Neighbor Embedding (t-SNE) is a nonlinear dimensionality reduction technique commonly used for visualization of high-dimensional data in a low-dimensional space, usually two or three dimensions. This method is aimed at preserving the intrinsic geometry of the relations between samples, while allowing for a visual interpretation of complex feature spaces.

t-SNE converts pairwise similarities between high-dimensional samples into probability distributions and minimizes the divergence between high-dimensional and low-dimensional representations. The method is particularly effective at preserving local neighborhood structures, making it suitable for revealing cluster patterns and local relationships among samples. However, t-SNE may distort global distances and is computationally expensive for large datasets.

**Figure 8:**
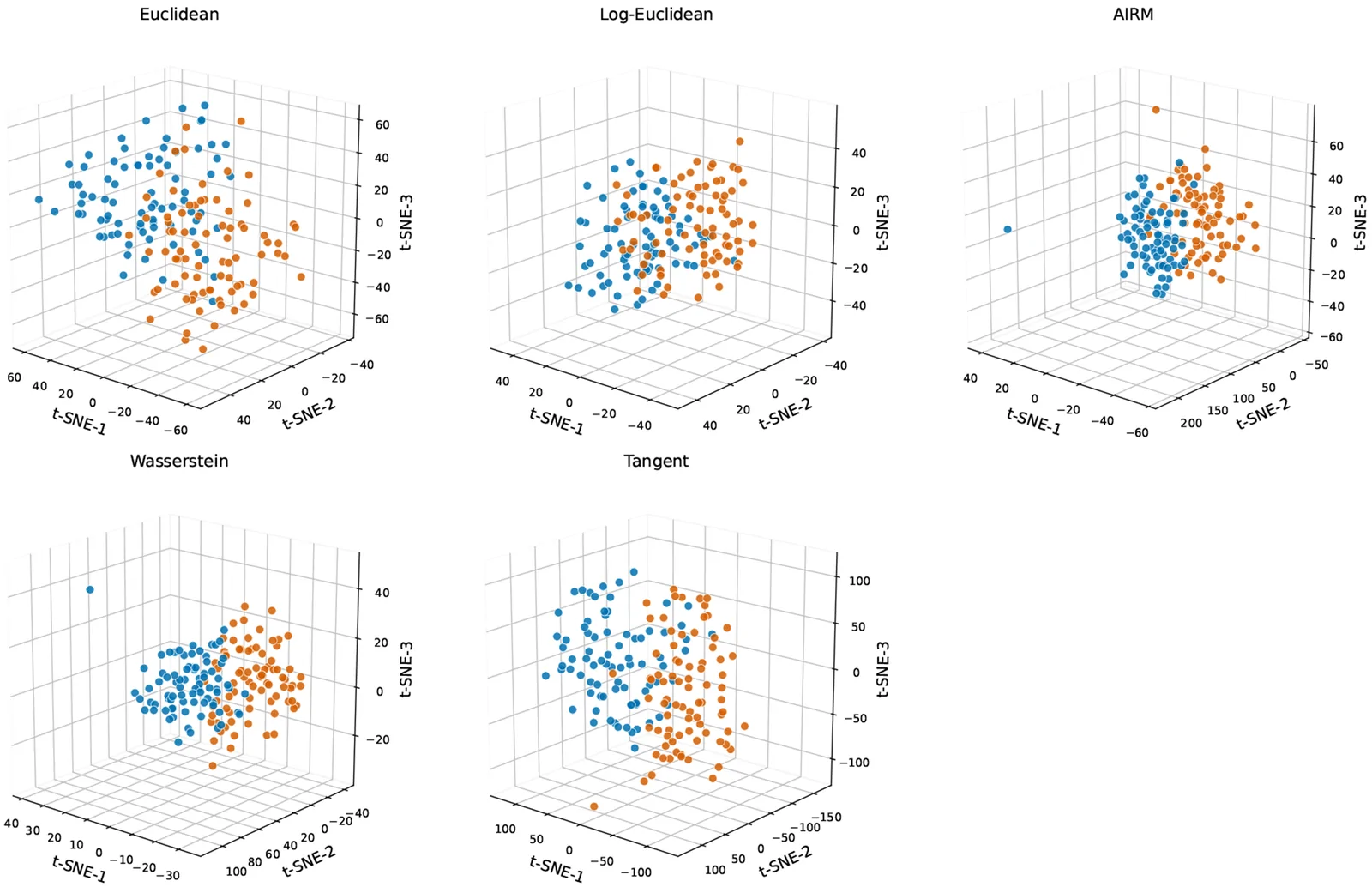
3D t-SNE visualization of the learned SPD representations under Euclidean, Log-Euclidean, AIRM, Wasserstein and Tangent Space metrics. Each point represents one subject, blue and orange points represent cognitively normal (CN) and Alzheimer’s disease (AD) subjects, respectively. The embeddings show the class separation obtained by each metric.

In this study, we used t-SNE to visualize the discriminative ability of the extracted SPD manifold features by investigating the separability between Alzheimer’s disease and healthy control subjects in the learned feature space. While the SPD data embedded in a 3D space are not strongly separable (see Figure 8), this is expected due to the intrinsic geometric structure of the data. The proposed features are defined on a Symmetric Positive Definite (SPD) manifold instead of a Euclidean space, and thus the discriminative information is embedded in the underlying Riemannian geometry.

### 3.4 Comparison

Zeng et al. [3] proposed a Teichmüller shape descriptor based on Ricci flow and used an SVM classifier for comparison with existing methods, and obtained an accuracy of 91.38% on the ADNI dataset. Shi et al. [4] used Ricci flow to propose a hyperbolic Wasserstein distance in the diagnosis of Alzheimer’s disease and achieved an accuracy of 76%. Acharya et al. [17] suggested Shearlet transform with KNN classifier and got 94.54% accuracy on ADNI dataset. Abbas et al. [18] used a Jacobian map with a CNN based model for Alzheimer’s disease diagnosis and achieved an accuracy of 96.61%. In our previous work [8], we fused the covariance descriptors obtained from Ricci energy optimization and a KNN classifier, the accuracy was 96%. In another study [19], conformal descriptors with Ricci flow parameterization and XGBoost classifier achieved 96.88% accuracy on the ADNI dataset. Moreover, in our previous work [14], descriptors of area distortion, conformal factor and Gaussian curvature from Ricci flow were evaluated with multiple classifiers such as MLP, XGBoost, Random Forest (RF), SVM, KNN, Decision Tree (DT) and Logistic Regression (LR) leading to a maximum accuracy of 98.62% for Alzheimer’s disease diagnosis.

As another evaluation in the current study, the suggested SPD data were projected into appropriate feature spaces by different metrics and the obtained representations were classified by the Support Vector Machine (SVM), Logistic Regression (LR), and Extreme Gradient Boosting (XGBoost). The results shown in Table 2. The XGBoost classifier with Wasserstein metric mapping, after KNN with AIRM (96%) and Log-Euclidean (94%) achieved an accuracy of 88%. This analysis provides a detailed comparison of the proposed framework in particular the KNN classifier with the AIRM metric in terms of distance based and traditional machine learning classifiers.

## 4 Discussion

The experimental results demonstrate that the proposed geometry-aware framework effectively captures discriminative hippocampal structural changes associated with Alzheimer’s disease. By integrating discrete Ricci flow with covariance-based SPD representations, the method provides a meaningful way to model the progressive geometric deformation of hippocampal surfaces during conformal parameterization.

Among the evaluated manifold-aware metrics, the Affine-Invariant Riemannian Metric (AIRM) achieved the best classification performance, indicating that preserving the intrinsic geometry of SPD matrices is crucial for accurate similarity measurement. The Log-Euclidean and Wasserstein metrics also showed competitive performance, confirming the importance of Riemannian-based analysis. In contrast, the Euclidean metric produced weaker results, highlighting the limitations of treating covariance matrices as ordinary Euclidean data.

The visualization result using t-SNE showed limited linear separability, which further supports the assumption that the discriminative information is embedded in the non-Euclidean manifold structure rather than in standard Euclidean feature space. This emphasizes the necessity of geometry-aware classification methods for such data. This observation is further confirmed by the classification results obtained with the SVM, XGBoost, and Logistic Regression classifiers.

## 5 Conclusion

In this paper, we propose a geometry-aware framework for Alzheimer’s disease classification based on the analysis of the hippocampal surface using discrete Ricci flow and SPD manifold learning. It extracts descriptors of Heat Kernel Signature, Area Distortion and Conformal Factor at several Ricci energy optimization steps using the geometric evolution of hippocampal surfaces during Ricci flow optimization. These descriptors were modeled by covariance matrices leading to a sequence of SPD representations for each subject.

To preserve the intrinsic manifold geometry, a KNN classification framework was used with several SPD-aware metrics. Experimental results on the ADNI dataset show that the best classification performance is achieved by the Affine-Invariant Riemannian Metric (AIRM) and Log-Euclidean metrics, which outperform the other metrics under evaluation. In addition, another classification with SVM, XGBoost, and Logistic Regression showed that the manifold-aware learning can provide more discriminative and geometrically meaningful representation than the conventional Euclidean analysis.

Future work will involve the integration of more geometric descriptors, the generalization of the framework for multi-modal neuroimaging data, and the investigation of deep Riemannian learning models to improve classification performance.

## Figures and Tables

**Table 1:** Demographic characteristics of the study participants, including the number of subjects, sex distribution, age statistics.

Class	Subjects	Age Range (years)	Male/Female
Alzheimer’s Disease (AD)	100	61–89	ADNI-1 (30/20) , ADNI-2 (28/22)
Cognitively Normal (CN)	100	60–88	ADNI-1 (27/23) , ADNI-2 (29/21)

**Table 2:** Performance in classification of machine learning classifiers and different SPD matrix representations. The best result for each evaluation metric is highlighted in bold.

Representation	Classifier	Accuracy	Precision	Recall	F1-Score
Euclidean	KNN	**0.82** ± **0.05**	**0.78** ± **0.03**	**0.88** ± **0.04**	**0.83** ± **0.05**
	SVM	0.78 ± 0.04	0.72 ± 0.05	0.92 ± 0.03	0.81 ± 0.04
	Logistic Regression	0.72 ± 0.03	0.68 ± 0.02	0.84 ± 0.03	0.75 ± 0.02
	XGBoost	**0.82** ± **0.03**	**0.81** ± **0.04**	**0.84** ± **0.03**	**0.82** ± **0.05**

Log-Euclidean	KNN	**0.94** ± **0.03**	**0.92** ± **0.05**	**0.96** ± **0.04**	**0.94** ± **0.06**
	SVM	0.86 ± 0.05	0.85 ± 0.04	0.88 ± 0.06	0.86 ± 0.05
	Logistic Regression	0.68 ± 0.05	0.67 ± 0.03	0.72 ± 0.03	0.69 ± 0.04
	XGBoost	0.82 ± 0.04	0.81 ± 0.05	0.84 ± 0.04	0.82 ± 0.03

AIRM	KNN	**0.96** ± **0.02**	**0.96** ± **0.02**	**0.96** ± **0.03**	**0.96** ± **0.02**
	SVM	0.86 ± 0.03	0.85 ± 0.04	0.88 ± 0.03	0.86 ± 0.02
	Logistic Regression	0.68 ± 0.03	0.67 ± 0.04	0.72 ± 0.05	0.69 ± 0.04
	XGBoost	0.82 ± 0.04	0.81 ± 0.03	0.84 ± 0.01	0.82 ± 0.02

Wasserstein	KNN	0.72 ± 0.02	0.65 ± 0.02	0.92 ± 0.01	0.76 ± 0.02
	SVM	0.74 ± 0.02	0.70 ± 0.03	0.84 ± 0.04	0.76 ± 0.02
	Logistic Regression	0.72 ± 0.04	0.70 ± 0.02	0.76 ± 0.02	0.73 ± 0.03
	XGBoost	**0.88** ± **0.05**	**0.88** ± **0.03**	**0.88** ± **0.02**	**0.88** ± **0.03**

Tangent Space	KNN	0.82 ± 0.03	0.80 ± 0.01	0.84 ± 0.02	0.82 ± 0.04
	SVM	0.70 ± 0.05	0.71 ± 0.03	0.68 ± 0.02	0.69 ± 0.04
	Logistic Regression	0.72 ± 0.03	0.76 ± 0.02	0.64 ± 0.01	0.70 ± 0.03
	XGBoost	**0.86** ± **0.03**	**0.91** ± **0.01**	**0.91** ± **0.01**	**0.85** ± **0.02**

## Data Availability

The data that support the findings of this study are available from the Alzheimer’s Disease Neuroimaging Initiative (ADNI) database (https://adni.loni.usc.edu). Access to the data is available to qualified researchers upon application and approval by ADNI.
